# Detection of KRAS mutation via ligation-initiated LAMP reaction

**DOI:** 10.1038/s41598-019-42542-x

**Published:** 2019-04-11

**Authors:** Yixin Fu, Xiaolei Duan, Jian Huang, Lizhen Huang, Lutan Zhang, Wei Cheng, Shijia Ding, Xun Min

**Affiliations:** 1 Department of Laboratory Medicine, The Affiliated Hospital of Zunyi Medical University, Zunyi, 563003 P.R. China; 2 School of Laboratory Medicine, Zunyi Medical University, Zunyi, 563003 P.R. China; 3 Key Laboratory of Clinical Laboratory Diagnostics (Ministry of education), College of Laboratory Medicine, Chongqing Medical University, Chongqing, 400010 P.R. China; 4 The Center for Clinical Molecular Medical Detection, The First Affiliated Hospital of Chongqing Medical University, Chongqing, 400010 P.R. China

## Abstract

KRAS mutations are abnormalities widely found in genomic DNA and circulating tumor DNA (ctDNA) of various types of cancers. Thus, highly sensitive detection of KRAS mutations in genomic DNA is of great significance in disease diagnosis and personalized medicine. Here, we developed a ligation-initiated loop-mediated isothermal amplification (LAMP) assaying method for ultrasensitive detection of KRAS mutation. In the presence of mutant KRAS DNA (mutDNA), the dumbbell-shaped structure (DSS) is formed by the specific ligation of two substrates (SLS1 and SLS2), which act as a template to initiate the following LAMP amplification. Making use of the outstanding specificity of ligation reaction and superior amplification of LAMP, 10 aM mutDNA can be accurately determined. In addition, as low as 0.1% mutDNA can be detected in the presence of a large excess of wild-type KRAS DNA (wtDNA), indicating the high sensitivity and specificity of the method. Furthermore, this strategy has been successfully applied for detection of a KRAS mutation from tissue samples of colorectal cancer patients. Thus, the developed ligation-initiated LAMP fluorescence assaying strategy presents a promising prospect for ultrasensitive detection of mutations.

## Introduction

Single nucleotide mutation is the permanent variation in a single nucleotide among the genomic DNA, thus altering the level of gene expression and linking to disease development^1–3^. As well known, the KRAS gene is a proto-oncogene which mainly participates in RAS-MAPK signaling pathway to control cell growth, proliferation and differentiation^4–6^. Moreover, single nucleotide mutation often occurs in and near codons of the KRAS gene, causing normal cells to become cancerous state^7,8^. Whole genome sequencing indicates that KRAS mutations are activated in various malignancies, such as colorectal cancer^9^, non-small-cell lung cancer^10,11^ and pancreatic adenocarcinoma^12^. Importantly, as a potential personalized biomarker, KRAS mutations can be used to not only predict the primary tumor but monitor minimal residual tumor during therapy^13,14^. Therefore, accurate and ultrasensitive detection of KRAS mutations will provide a robust evidence for early diagnosis of mutant KRAS-related cancer.

Various technology platforms have been applied to discriminate mutation, among which, DNA sequencing is a classic mutation detection technique that allows high-throughput screening of the individual nucleotides within a DNA sequence^15,16^. However, the sophisticated instrumentation and complicated procedures are the limiting factors for its extensive application^17^. The polymerase chain reaction (PCR)-based methods, such as wild-type blocking PCR^18^ and allele-specific PCR^19^, have received increasing attention owing to the advantages of simple assaying protocols and instrument automation, also these methods still require the careful design of specific probes for discrimination. Therefore, there is still an urgent need for the development of highly sensitive, cost-effective and rapid mutation detection strategy.

Currently, ligation reaction mediated by DNA ligase holds the capacity for characterization of mutation, fusion gene and other genetic alterations^20,21^. DNA ligase can efficiently catalyze the junction of the 3′-end of one DNA fragment with the 5′-end of another DNA fragment upon hybridization with a complementary DNA target^22^. Due to the excellent discrimination capacity of ligase, the ligation reaction demonstrates better specificity than primer extension reaction for single nucleotide polymorphisms^23–25^. For example, adopting the end-joining fidelity of DNA ligase on single nucleotide polymorphisms, a novel iLock assay with an improved specificity was developed for the detection of a mutation in mRNA^26^. Meanwhile, the various ligation based amplification strategies have been developed, which are extensively used to detect methylation and mutation at the single nucleotide level^27–29^. Thus, the ligation reaction mediated by DNA ligase can serve as a specific method to characterize single-base mutation in DNA strands with the merits of excellent discrimination capacity and simple detection process.

Loop-mediated isothermal amplification (LAMP), an outstanding isothermal amplification technique, has been proved to be a highly specific, sensitive and rapid detection method^30–32^. In LAMP reaction, owing to its high strand displacement activity, Bst DNA polymerase is widely used to efficiently amplify DNA sequences^33^, making LAMP suitable for point-of-care diagnostics. Recently, there have been a number of published upgrade strategies for LAMP, integrating LAMP with other DNA amplification techniques, such as PCR^34^, reverse transcription^35^ and ligation assays^36^. For instance, Du and coworkers have developed a ligation-LAMP strategy for the detection of miRNA with a low detection limit of 0.2 fM. With the upgrade LAMP strategies, the detection performance of LAMP changes from simplification to diversification, validating its potential as a promising technique in the application of diagnostic.

**Figure 1:**
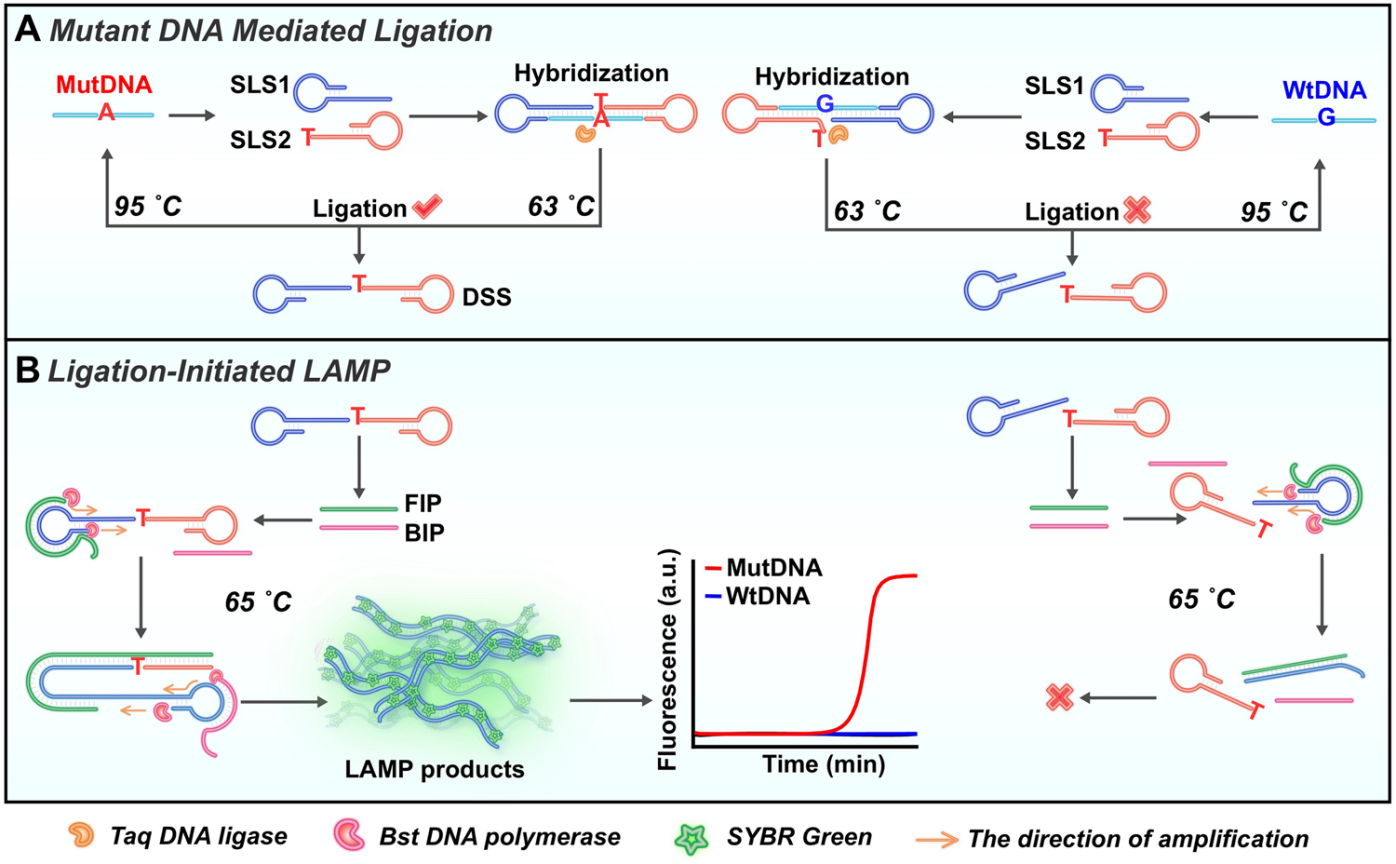
The principle of ligation-initiated LAMP strategy. (**A**) The formation of dumbbell-shaped structure (DSS) by mutDNA mediated ligation reaction. (**B**) LAMP reaction initiated by the DSS.

Herein, making use of the remarkable specificity of ligation reaction and highly amplified efficiency of LAMP, we developed a label-free ligation-initiated LAMP strategy for specific and sensitive detection of KRAS mutation. As shown in Fig. 1, the developed method mainly comprises two step reactions: the ligation reaction mediated by mutDNA and the ligation-initiated LAMP reaction. In the ligation reaction, two ligation substrates, stem-loop substrate 1 (SLS1) and stem-loop substrate 2 (SLS2), are designed for discriminating the single base between mutant KRAS DNA (mutDNA) and wild-type KRAS DNA (wtDNA) respectively. The SLS1 is completely complementary to mutDNA and wtDNA. While, compared with the mutDNA, there is one more position mismatch between wtDNA and SLS2 (Fig. S1, Supporting Information). In the presence of mutDNA, SLS1 and SLS2 can be ligated to form the dumbbell-shaped structure (DSS) by the catalysis of Taq DNA ligase at 63 °C. Meanwhile, the base T at the 3′-end of the SLS2 is not complementary to the base G in the wtDNA owing to the one more position mismatch. Thus, no ligation occurs in the presence of wtDNA. The DSS is just the starting template for LAMP amplification. Along with the gradient temperature cycles, more DSSs are produced in the presence of mutDNA. In the second step, forward inner primer (FIP) first hybridizes with the loop in the DSS for strand displacement DNA synthesis. Meanwhile, self-primed DNA synthesis is initiated from the 3′ to 5′ by using self-structure of DSS as the template. With high-efficiency strand displacement activity, the Bst DNA polymerase initiates the amplification reaction and creates more elongated structures along the DSS. Then, backward inner primer (BIP) hybridizes to the elongated structures to initiate subsequent rounds of amplification reaction, generating a large amount of double strand DNA (dsDNA). As a result, a significant fluorescence signal is achieved by the preferential binding between the fluorescence dye SYBR Green I and dsDNA. On the contrary, without formation of DSS, there is a negligible fluorescence background from the detection of wtDNA. The developed strategy kept a low background signal for the detection of KRAS mutation by adding a mismatch site. Moreover, the developed strategy can also be successfully applied for the detection of KRAS mutation from tissue samples of colorectal cancer patients, presenting a promising application for ultrasensitive detection of mutation in clinical diagnosis.

## Results and Discussion

### The feasibility of the ligation-initiated LAMP

**Figure 2:**
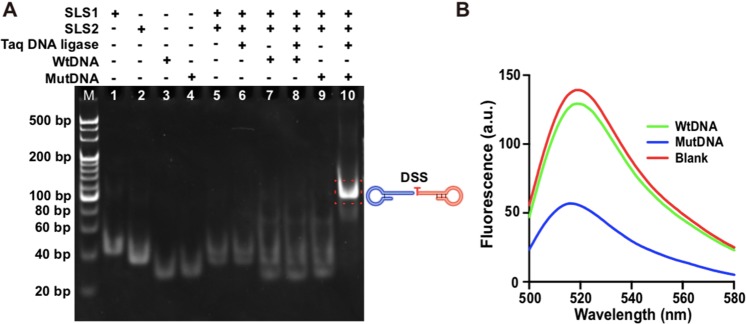
(**A**) PAGE analysis of the ligation products. 20 bp DNA marker, SLS1 (lane 1), SLS2 (lane 2), wtDNA (lane 3), mutDNA (lane 4), the mixture of SLS1 and SLS2 (lane 5), the mixture of SLS1 and SLS2 under ligation of Taq DNA ligase (lane 6), the mixture of SLS1, SLS2 and wtDNA (lane 7), the mixture of SLS1, SLS2 and wtDNA under ligation of Taq DNA ligase (lane 8), the mixture of SLS1, SLS2 and mutDNA (lane 9), the mixture of SLS1, SLS2 and mutDNA under ligation of Taq DNA ligase (lane 10). (**B**) Fluorescence spectra from the ligation reaction products mediated by blank, wtDNA and mutDNA, respectively.

**Figure 3:**
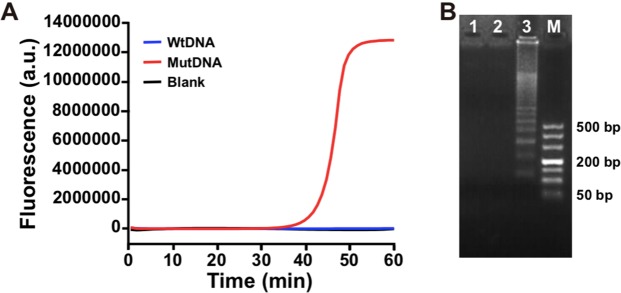
(**A**) Real-time fluorescence curves produced by 1 fM mutDNA (red line), 1 fM wtDNA (blue line) and blank (black line) with developed ligation-initiated LAMP strategy, respectively. (**B**) Gel electrophoresis analysis of 10 µL LAMP products: ligation products of blank (lane 1), wtDNA (lane 2), mutDNA (lane 3), (Lane M, DL500 DNA marker).

The feasibility of ligation reaction was firstly verified by 8% native PAGE. As shown in Fig. 2A, lanes 1–4 indicated the position of substrate SLS1, substrate SLS2, wtDNA and mutDNA, respectively. Lane 5 represented the mixture of SLS1 and SLS2. With addition of Taq DNA ligase to the mixture of SLS1 and SLS2, unchanged electrophoretic bands (lane 6) were achieved compared to lane 5, indicating the ligation reaction did not take place between SLS1 and SLS2 without the complementary DNA template. With addition of the wtDNA (or mutDNA) to the mixture of SLS1 and SLS2, a small band with lower electrophoresis migration was observed in the lanes 7 and 9. Then, with the addition of Taq DNA ligase (lane 8), there was no difference in the products of wtDNA mediated ligation compared to lane 7, while there was a distinct band with much lower mobility appeared in the mutDNA mediated ligation reaction (lane 10), indicating the formation of ligation products (DSS). Further, we labeled a fluorophore on the 5′-end of SLS1-1, and a quencher at the 3′-end of SLS2-1 to further confirm the mutDNA mediated ligation reaction. As shown in Fig. 2B, the highest fluorescence intensity was obtained in the wtDNA mediated ligation reaction, which is comparable to the fluorescence intensity of the blank (without mutDNA or wtDNA). However, owing to the short distance between fluorophore and quencher after mutDNA mediated ligation, a low fluorescence intensity was obtained. These results proved that the ligation could only be achieved in the presence of mutDNA. Further, the ligation products were amplified and quantified by real-time fluorescence monitored LAMP. As shown in Fig. 3A, the fluorescence intensity raised sharply with increase of the time after 35 min and then reached a plateau within 60 min in the presence of mutDNA mediated ligation product. However, no apparent fluorescence signal change was observed in the presence of wtDNA mediated ligation product or blank sample, due to no formation of DSS. Meanwhile, agarose electrophoresis was adopted to analyze the products of LAMP (Fig. 3B). A ladder-like pattern bands emerged from the LAMP product initiated by mutDNA mediated ligation (Fig. 3B, lane 3), demonstrating the successful amplification of the LAMP to produce long dsDNA product with cauliflower-like structures. In contrast, there were no apparent bands for the LAMP product initiated by wtDNA mediated ligation (Fig. 3B, lane 2) and blank sample (Fig. 3B, lane 1). These results were in good agreement with the real-time fluorescence measurements, further validating the feasibility of the ligation-initiated LAMP strategy for mutDNA detection.

### Optimization of the experimental conditions

In order to achieve the optimal analytical performance, several experimental conditions were investigated. According to the recent reports, inserting an extra mismatch at certain positions of the probes could change the Tm of duplex, which has been verified to affect the discrimination between wild and mutant gene^37,38^. Therefore, an additional mismatch at different sites (2^nd^, 3^rd^, 4^th^, 5^th^, 6^th^) within SLS2 was designed (Table S1) to increase the allele-specificity ligation. The ligation product of SLS1 and mismatched SLS2 mediated by mutDNA or wtDNA was added to real-time monitored LAMP reaction, separately. Meanwhile, the POI value (point of inflection, represents the time corresponding to the maximum slope of fluorescence curves) was employed to quantitatively evaluate the amplification efficiency. The lowest POI_m_ POI_w_^−1^ ratio (POI_m_, POI of mutDNA; POI_w_, POI of wtDNA) could make the best distinction between mutDNA and wtDNA. As shown in Fig. S2, the lowest POI_m_ POI_w_^−1^ ratio was obtained from the additional mismatch at the 3^rd^ site. Thus, the 3^rd^ site mismatch within SLS2 was the best optimal mismatch site. The other experimental parameters, including the ligation temperature, cycle numbers of ligation reaction, the concentration of Bst DNA polymerase and the temperature of LAMP reaction, were also investigated (Figs S3–S6). As a result, the optimal ligation temperature and cycle number were 63 °C and 30 cycles, respectively. The concentration of Bst DNA polymerase was optimized to be 0.4 U µL^−1^, and optimal temperature of ligation-initiated LAMP was 65 °C.

### Analytical performance of the ligation-initiated LAMP strategy in synthetic mutDNA

**Figure 4:**
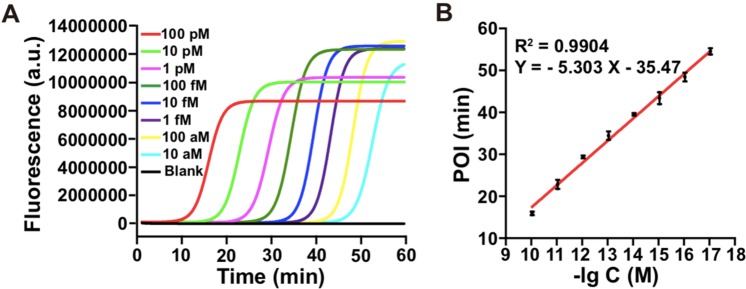
(**A**) Real-time fluorescence measurement of different concentrations (100 pM, 10 pM, 1 pM, 100 fM, 10 fM, 1 fM, 100 aM, 10 aM and 0) of mutDNA based on ligation-initiated LAMP strategy. (**B**) The linear relationship between POI (Y) and the logarithm of mutDNA concentrations (X). Error bars show the standard deviation of three replicative tests.

Under the optimal experimental conditions, the analytical performance of the developed ligation-initiated LAMP method was evaluated by real-time fluorescence monitor of different concentrations of synthetic mutDNA. As shown in Fig. 4A, the POI values were gradually shortened with the increasing concentration of mutDNA in the range from 10 aM to 100 pM. As illustrated in Fig. 4B, the POI value (Y) exhibited a good linear correlation with the logarithm of mutDNA concentrations (X) in the range from 10 aM to 100 pM, and the correlation equation was Y = −5.303X −35.47 with R^2^ = 0.9904. The developed strategy had a wide linear dynamic range over 7 orders of magnitude, and down to 10 aM target DNA could be detected in 60 min. Compared with the reported methods ligation-based strand displacement amplification and ligation-based rolling circle amplification (Supporting Information, Table S2), the ultra-sensitivity achieved for detection of mutDNA can be ascribed to the highly efficient signal amplification of LAMP, which generates upwards of 10^9^ copies from less than 100 copies of target DNA within one hour^39^.

### Specificity of the ligation-initiated LAMP strategy in synthetic DNA strands

**Figure 5:**
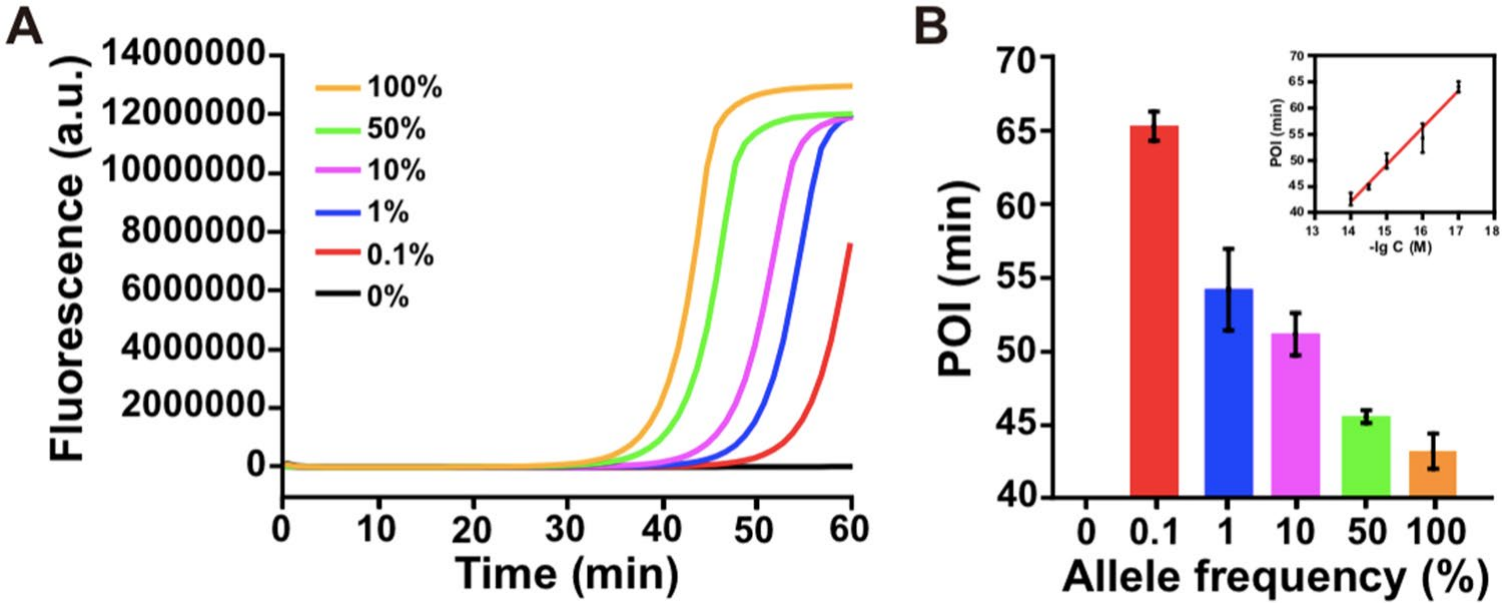
Detection of KRAS mutation with different proportions between mutDNA and wtDNA. (**A**) Real-time fluorescence curves of ligation-initiated LAMP reaction produced by a mixture of target DNA with different ratios of mutDNA to the total DNA (allele frequency). The allele frequency was 0%, 0.1%, 1%, 10%, 50% and 100%, and the total DNA concentration was 10 fM. (**B**) The relationship between the POI values from (**A**) and allele frequency. The error bars represent standard deviation of three replicate measurements.

To test the specificity of the developed method for effective discrimination of trace mutDNA, the wtDNA and mutDNA were mixed with a total concentration of 10 fM, in which the ratio of mutDNA to the total DNA varying from 0% to 100%. Afterwards, the mixed samples were assayed, and the corresponding real-time fluorescence curves were monitored. As shown in Fig. 5A, along with the decreasing concentration of mutDNA, the POI values were significantly increased (Fig. 5B). As a result, even 0.1% mutDNA could be clearly discriminated from that of 10 fM wtDNA, which was much lower than those reported mutation detection strategies (Supporting Information, Table S2). These strategies mainly depend on the 3’ terminal nucleotide of a primer that corresponds to a specific mutation site to discriminate mutation, whereas it is not sufficient to achieve reliable discrimination between the alleles. Therefore, according to the combination of rational probe design and high amplification efficiency of LAMP, the strategy achieved a high specificity and superior sensitivity for mutation detection in DNA strand.

### Detection of the KRAS mutation from tissue samples

**Figure 6:**
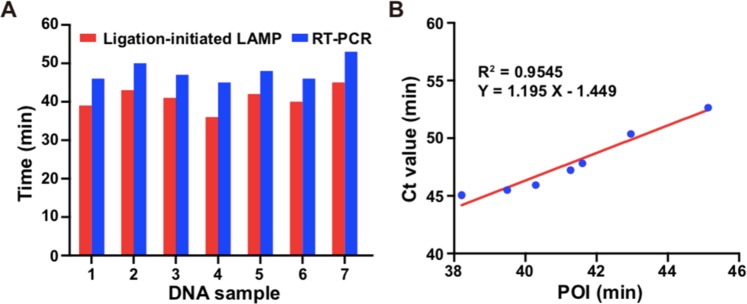
Detection of KRAS mutation in genomic DNA from tissue samples. (**A**) Histogram and (**B**) correlation plot of POI value of proposed strategy and Ct value of RT-PCR.

Finally, the genomic DNA extracted from tissue samples of ten colorectal cancer patients were assayed by the developed strategy to evaluate the potential application. As a result, 7 of 10 samples harbored the KRAS mutation, whereas the other 3 samples were negative. The result was consistent with the result obtained from the commercial RT-PCR qualitative kit approved by the China Food and Drug Administration (CFDA), indicating that the developed strategy could discriminate between negative and positive tissue samples. Figure 6A showed the mean POI value and corresponding time of cycle threshold (Ct, RT-PCR) of seven positive samples. The relationship between the results of RT-PCR and the ligation-initiated LAMP strategy was further investigated by regression analysis of the corresponding time of Ct and the mean value of POI. As shown in Fig. 6B, the regression equation was Y = 1.195X −1.449 with R^2^ = 0.9545, indicating there was a good correlation between the two methods. These results demonstrated the proposed strategy can be applied for the identification of KRAS mutation in genomic DNA from tissue samples, holding a great potential in the diagnosis of mutation-related human diseases.

## Conclusion

In summary, in combination with ligation and LAMP, a novel fluorescent sensing strategy has been developed for detection of DNA mutation. Owing to rational probe design by adding an extra mismatch to SLS2 and efficient amplification of LAMP, this ligation-initiated LAMP strategy not only achieves an ultrahigh sensitivity and specificity for detection of mutDNA at aM level but also can quantitate the rare mutation even in a large excess of coexisting wtDNA with a selectivity factor down to 0.1% in synthetic DNA strands. Moreover, the strategy has been successfully applied for the accurate detection of mutation from the tissue samples of colorectal cancer patients, and the results of our method are consistent with the that of RT-PCR. Therefore, the ligation-initiated LAMP strategy holds great potential application for the detection of DNA mutation, and also offers a new insight into disease diagnostics, personalized medicine and biomedical research.

## Experiment Section

### Materials and reagents

The DNA oligonucleotides were synthesized by Sangon Biotech Co., Ltd. (Shanghai, China) and purified by high-performance liquid chromatography. Taq DNA ligase (40 U μL^−1^), 10 × Taq DNA ligase reaction buffer (200 mM Tris-HCl, 250 mM KAc, 100 mM Mg(Ac)_2_, 100 mM DTT, 10 mM NAD, 1% Triton X-100, pH 7.6), Bst 2.0 DNA polymerase (8 U μL^−1^) and 10 × ThermoPol reaction buffer (200 mM Tris-HCl, 100 mM (NH_4_)_2_SO_4_, 500 mM KCl, 20 mM MgSO_4_, 1% Tween-20, pH 8.8) were all purchased from New England Biolabs (Beijing, China). SYBR Green I was obtained from Generay Biotech. Co., Ltd. (Shanghai, China). DNA marker, 6 × loading buffer and dNTPs were purchased from Takara (Dalian, China). TIANamp Genomic DNA Kit was purchased from Tiangen Biotech Co., Ltd. (Beijing, China). The nuclease-free water was purchased from Thermo Fisher Scientific Inc (Vilnius, Lithuania) and used in all ligation reaction and ligation-initiated LAMP assays. All the reagents were of analytical grade and used without further purification. All solutions were prepared with ultrapure water from Millipore Milli-Q water purification system (Millipore, USA). The detailed oligonucleotide sequences were listed in Table S1 (Supporting Information).

### Ligation reaction

The ligation mixture contained 1 µL Taq DNA ligase reaction buffer, 1 U µL^−1^ Taq DNA ligase, 100 nM SLS1 and 100 nM SLS2. For the detection of different target, various concentrations of mutDNA, wtDNA or genomic DNA was added to the ligation mixture to yield a final volume of 10 µL. Then the reaction mixture was subjected to the following thermal cycling profile in a thermal cycler (Bio-Rad, USA): initial denaturation at 95 °C for 3 min, 30 cycles at 95 °C for 0.5 min and 63 °C for 1 min. Then, another denaturation at 95 °C for 5 min was carried out, followed by quickly cooled down to 4 °C. The ligation reaction products were subjected to LAMP reaction.

### LAMP reaction and real-time fluorescence measurement

The LAMP reaction buffer contained 200 nM FIP, 200 nM BIP, and 0.25 mM dNTPs and 2 µL ThermoPol reaction buffer. For the detection of different ligation products, 1 µL mutDNA or wtDNA mediated ligation reaction products was added to the LAMP buffer with a total volume of 18 µL. Then the reaction mixture was heated to 95 °C for 5 min, followed by cooling at 4 °C for 5 min. After that, 1 µL SYBR Green I and 0.4 U µL^−1^ Bst 2.0 DNA polymerase were added. The reaction was performed at 65 °C in a StepOne Real-Time PCR instrument (Applied Biosystems, USA) for 60 min. The real-time fluorescence intensity was monitored at an interval of 1 min employing the SYBR Green channel.

### Gel electrophoresis

The products of ligation reaction were analyzed by 8% native polyacrylamide gel electrophoresis (PAGE) in 1 × TBE buffer (89 mM Tris, 89 mM boric acid, 2 mM EDTA) at a 90 V constant voltage for 45 min. The products of LAMP reaction obtained were analyzed by 2% agarose gel in 1 × TBE buffer at 110 V for 35 min. After gold view (GV) staining, the gels were photographed with a gel imaging system (Bio-Rad, USA).

### Genomic DNA extraction and purification from clinical samples

Samples and clinical data were collected from ten colorectal cancer patients at the first affiliated hospital of Chongqing Medical University with informed consent. Among which, seven samples were positive and three were negative. Genomic DNA from formalin-fixed, paraffin-embedded (FFPE) tissue samples was extracted by TIANamp Genomic DNA Kit according to the manufacture’s instruction, and stored at −80 °C until use, and the final concentration of genomic DNA was 0.4 ng µL^−1^. The DNA concentration was determined by NanoDrop One Microvolume UV-vis spectrophotometer (Thermo Scientific, USA).

## Supplementary information

Detection of KRAS mutation via ligation-initiated LAMP reaction
