# Supplementary material for: Detection of KRAS mutation via ligation-initiated LAMP reaction
Source: Sci Rep. 2019 Apr 11;9:5955. doi: 10.1038/s41598-019-42542-x (PMC6459849; doi:10.1038/s41598-019-42542-x)
Supplement: Supplementary file 1 — Detection of KRAS mutation via ligation-initiated LAMP reaction [file 41598_2019_42542_MOESM1_ESM.docx]

Electronic Supplementary Information for

**Detection of KRAS Mutation via** **Ligation-Initiated LAMP Reaction**

Yixin Fu^1,2,#^, Xiaolei Duan^2,3,#^, Jian Huang^1,2^, Lizhen Huang^4^, Lutan Zhang^4^, Wei Cheng^4^, Shijia Ding^3^, Xun Min^1,2,^*

^1^ Department of Laboratory Medicine, The Affiliated Hospital of Zunyi Medical University, Zunyi 563003, P.R. China.

^2^ School of Laboratory Medicine, Zunyi Medical University, Zunyi 563003, P.R. China.

^3^ Key Laboratory of Clinical Laboratory Diagnostics (Ministry of education), College of Laboratory Medicine, Chongqing Medical University, Chongqing 400010, P.R. China.

^4^ The Center for Clinical Molecular Medical Detection, The First Affiliated Hospital of Chongqing Medical University, Chongqing 400010, P.R. China.

^#^ These authors contributed equally to this work.

* Corresponding authors: Phone/Fax：0851-28608182.

E-mail: minxunzmu@163.com (X. Min).


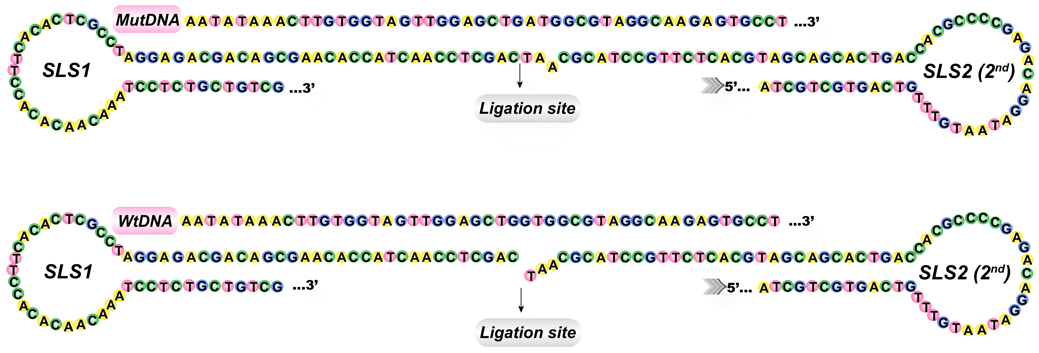


**Figure S1.** Schematic representation of ligation reaction for mutDNA and wtDNA.


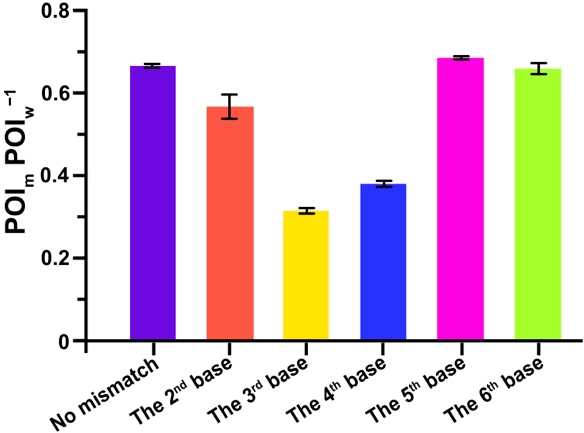


**Figure S2.** The effect of different mismatched sites within SLS2 on the specific detection of mutDNA. POI_m_ POI_w_^−1^ was the POI ratio of mutDNA to wtDNA. The concentrations of mutDNA and wtDNA were 1 pM. Other experimental conditions were the same as described in the experimental procedure.

**Optimization of the ligation temperature**

Ligation reaction consists of two steps: denaturation and ligation. The ligation temperature plays an important role in DSS formation, which greatly influences the efficiency of LAMP. So, the ligation temperature was investigated. Firstly, in the presence of blank, 1 fM wtDNA (as negative control) and different concentrations of mutDNA, ligation reaction was performed at various ligation temperature (55 ºC, 57 ºC, 63 ºC and 65 ºC), respectively. Then, the different ligation products were conducted by LAMP to evaluate the effect of ligation temperature on the detection of mutDNA. As shown in Fig. S3, all the products obtained by the mutDNA mediated ligation could initiate LAMP reaction, producing different fluorescence curves. While, the fluorescence curves for blank or wtDNA were a straight line near zero, suggesting there was no amplification product produced by LAMP. At 55 ºC, the POI value for 10 aM mutDNA was not obtained before the end of LAMP reaction. Meanwhile, along with the increased ligation temperature, the POI values for identical concentration of mutDNA were decreased, indicating the best efficiency of LAMP could be achieved at 65 ºC. However, at 65 ºC, the resolution of the fluorescence curves corresponding to adjacent concentration was too low, which could not effectively discriminate the higher concentration of mutDNA between 1 fM and 100 aM. Therefore, 63 ºC was selected as the optimized ligation temperature in the ligation reaction.


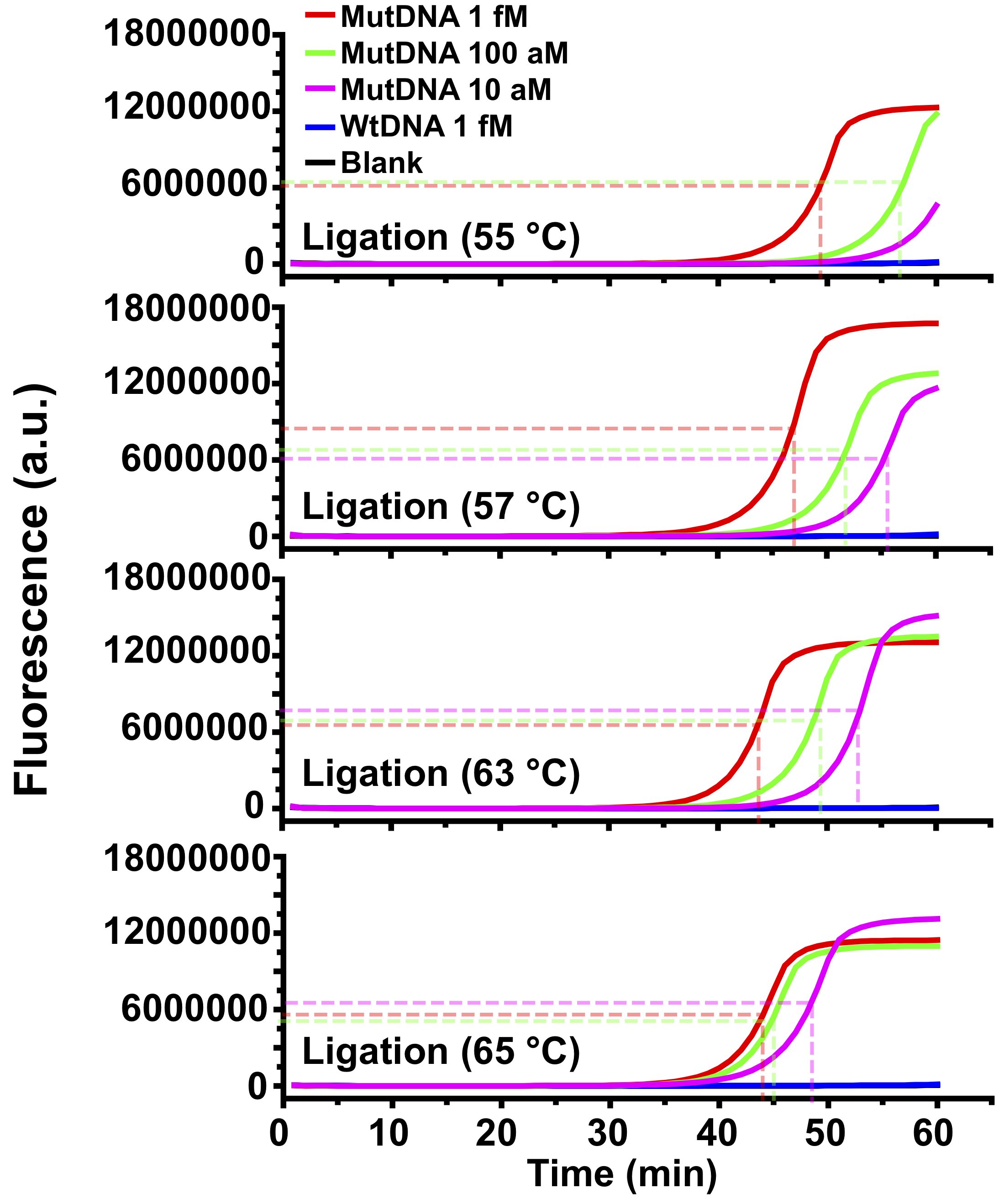


**Figure S3.** The real-time fluorescence curves for blank, wtDNA (1 fM), mutDNA (1 fM, 100 aM, 10 aM) at different ligation temperatures. Other experimental conditions were the same as described in the experimental procedure.

**Optimization of the ligation cycle number**

The cycle number of ligation reaction was optimized due to its effect on the production of DSS. After different ligation cycle, the targets (blank, wtDNA and mutDNA) mediated ligation products were detected by ligation-initiated LAMP. As shown in Fig. S4, only the mutDNA could induce the related fluorescence curves. And the POI values decreased along with the increasing ligation cycle numbers. Obviously, the 40 cycles used in ligation reaction contributed the lowest POI value, suggesting more DSS products could be formed and amplified by LAMP. Unfortunately, the difference of POI between 1 fM and 100 aM mutDNA was narrow, making a poor discrimination ability for the middle concentrations. Therefore, 30 cycles were employed for the ligation reaction assay.


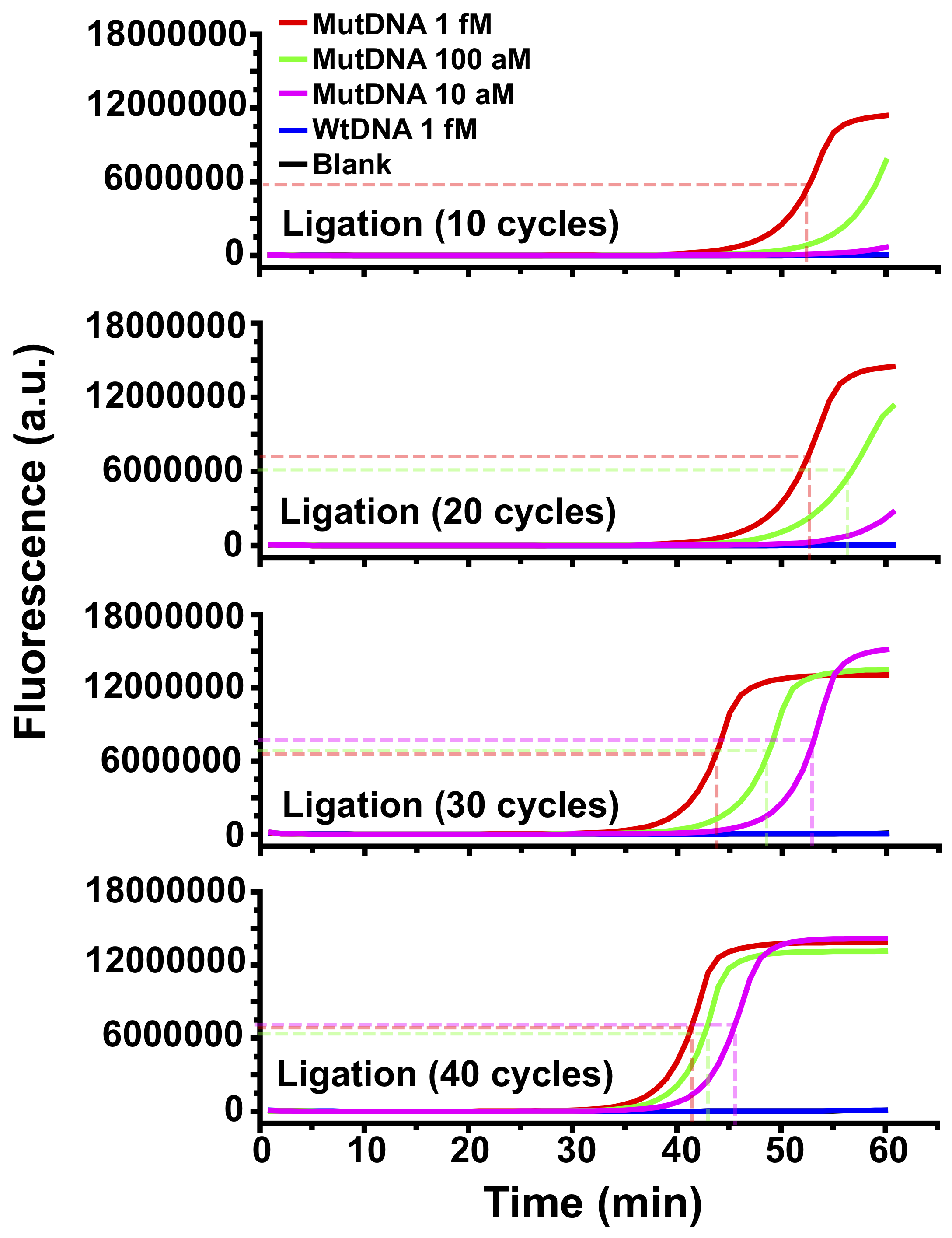


**Figure S4.** The real-time fluorescence curves for blank, wtDNA (1 fM), mutDNA (1 fM, 100 aM, 10 aM) at different ligation cycle numbers. Other experimental conditions were the same as described in the experimental procedure.

**Optimized the concentration of Bst DNA polymerase**

The concentration of Bst DNA polymerase was further optimized. With the minimum Bst DNA polymerase (0.16 U µL^−1^) was added in the LAMP reaction, no detectable fluorescence signal was observed at any concentration of mutDNA (Fig. S5). Then, with increasing concentration of Bst DNA polymerase in the LAMP reaction, the mutDNA could be well detected. While, when higher concentration of Bst DNA polymerase (0.52 and 0.64 U µL^−1^) was used in LAMP reaction, the fluorescence curves of blank and wtDNA significantly rose at the end of the reaction, indicating that there is a nonspecific amplification. Thus, taking the consideration of both reaction time and specificity, 0.4 U μL^−1^ Bst DNA polymerase was used in the LAMP reaction.


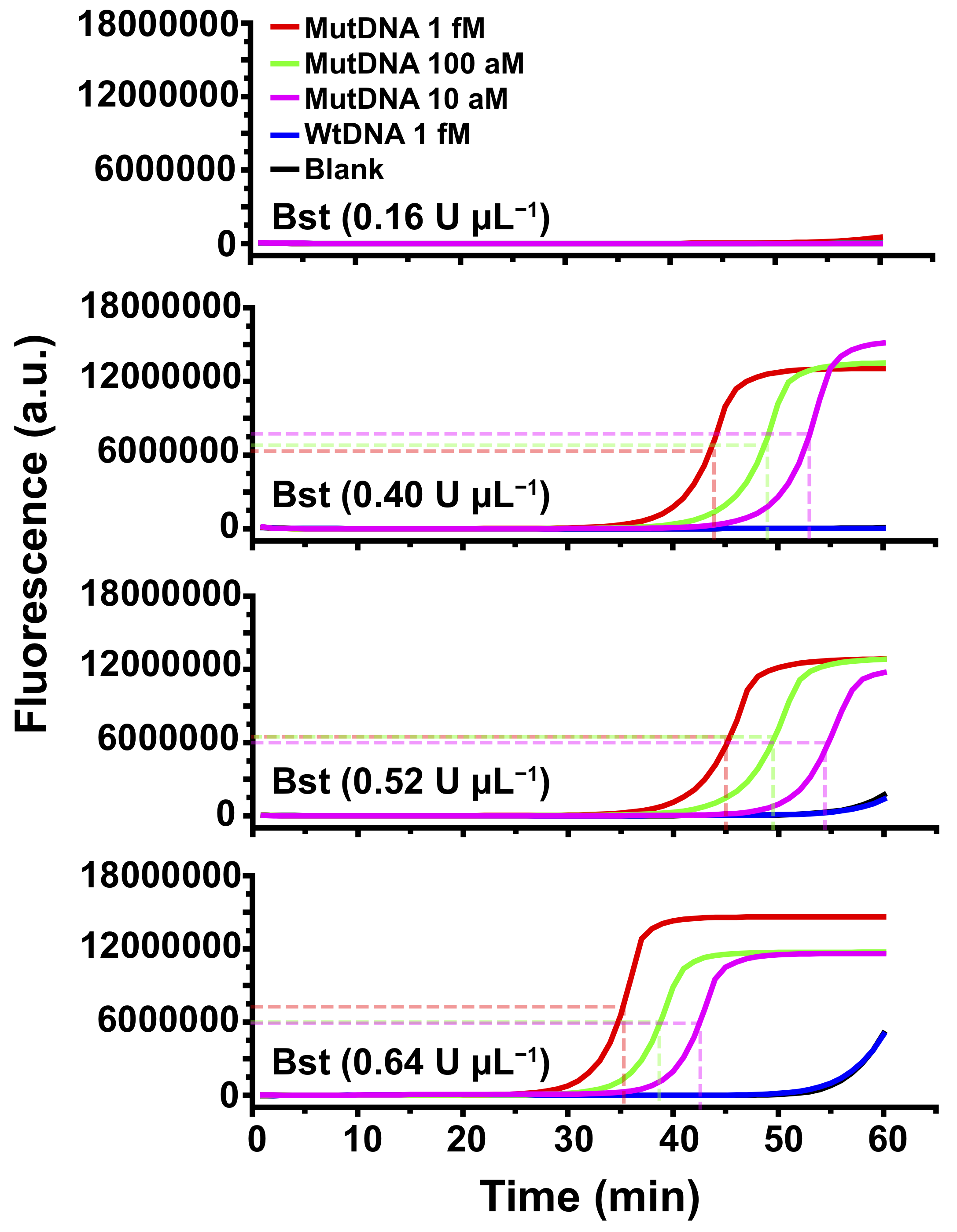


**Figure S5.** The ligation-initiated LAMP method for blank, wtDNA (1 fM), and mutDNA (1 fM, 100 aM, 10 aM) detection with addition of different concentration of Bst DNA polymerase (0.16, 0.4, 0.52 and 0.64 U µL^−1^). Other experimental conditions were the same as described in the experimental procedure.

**Optimization of the LAMP temperature**

The different reaction temperature of LAMP was also investigated by detecting the real-time fluorescence curves of blank, mutDNA (10 aM, 100 aM, 1 fM) and wtDNA (1 fM), respectively. As shown in Fig. S6, negligible fluorescence signal was observed for 10 aM mutDNA at 60 ºC. Meanwhile, when the temperature reached up to 70 ºC, no detectable signals were obtained at different concentrations. Significantly, mutDNA at different concentrations could be well detected at 65 ºC. Therefore, 65 ºC was chosen for the optimum temperature of LAMP.


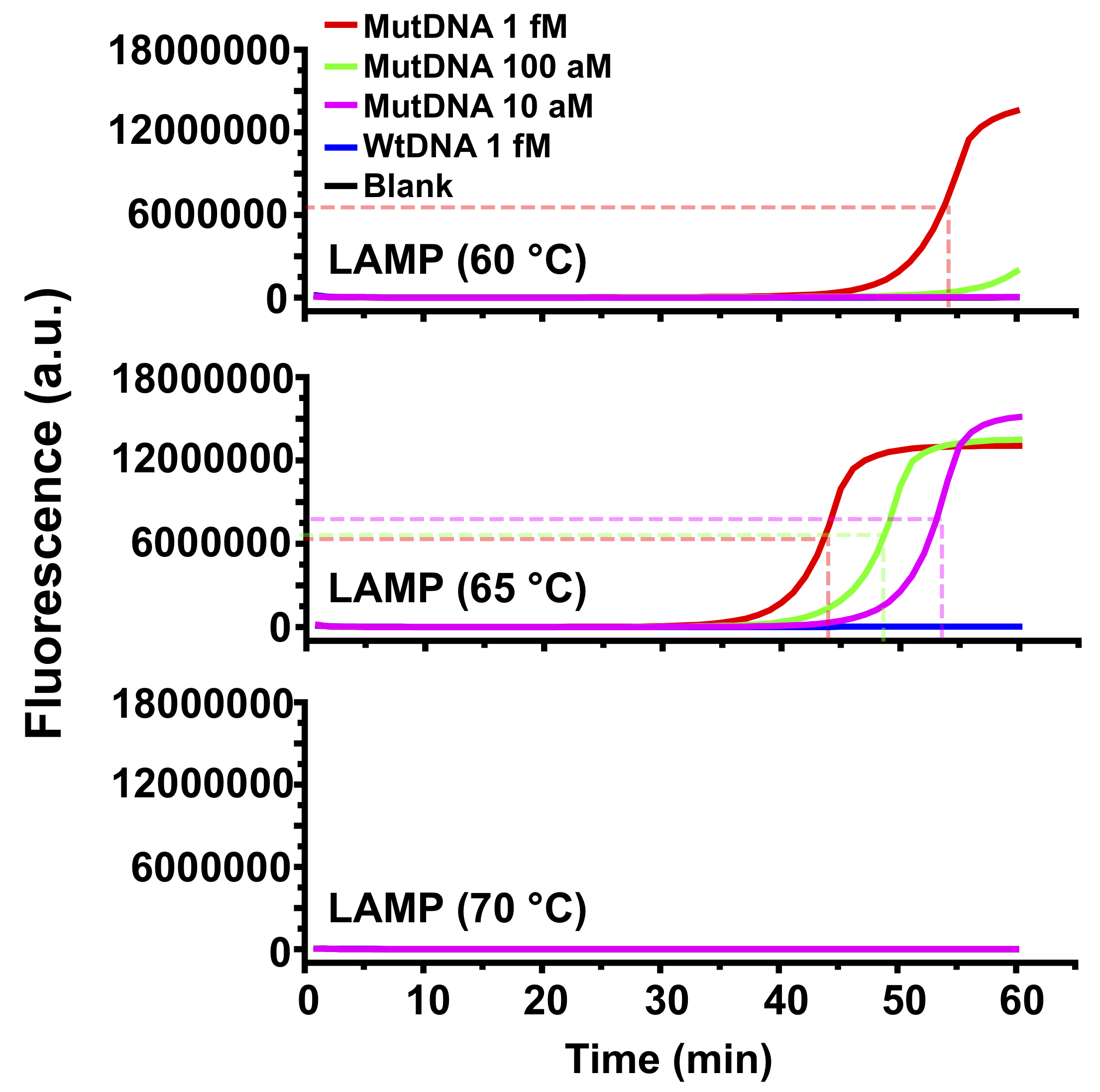


**Figure S6.** The real-time fluorescence curves for blank, wtDNA (1 fM), mutDNA (1 fM, 100 aM, 10 aM) at different temperature of (60 ºC, 65 ºC and 70 ºC), respectively. Other experimental conditions were the same as described in the experimental procedure.

**Table S1.** The sequences of oligonucleotides used in this work.

| **Name** | **Sequence (5’ – 3’)** |
| --- | --- |
| SLS1 | phosphate-CAGCTCCAACTACCACAAGCGACAGCAGAGGATCCGCTCACACTTCCACACAACAAATCCTCTGCTGTCG |
| SLS2 | ATCGTCGTGACTGTTTGTAATAGGACAGAGCCCCGCACCAGTCACGACGATGCACTCTTGCCTACGCCAT |
| FIP | CGACAGCAGAGGATTTGTTGTGTGGAAGTGTGAGCGG |
| BIP | ATCGTCGTGACTGTTTGTAATAGGACAGAGCCCCGCAC |
| MutDNA | AATATAAACTTGTGGTAGTTGGAGCTG**A**TGGCGTAGGCAAGAGTGCCT |
| WtDNA | AATATAAACTTGTGGTAGTTGGAGCTGGTGGCGTAGGCAAGAGTGCCT |
| SLS1-1 | phosphate-CAGCT(FAM)CCAACTACCACAAG |
| SLS2-1 | GCACTCTTGCCTACGCAAT(BHQ1) |
| SLS2 (2^nd^) | ATCGTCGTGACTGTTTGTAATAGGACAGAGCCCCGCACCAGTCACGACGATGCACTCTTGCCTACGCCCT |
| SLS2 (3^rd^) | ATCGTCGTGACTGTTTGTAATAGGACAGAGCCCCGCACCAGTCACGACGATGCACTCTTGCCTACGCAAT |
| SLS2 (4^th^) | ATCGTCGTGACTGTTTGTAATAGGACAGAGCCCCGCACCAGTCACGACGATGCACTCTTGCCTACGACAT |
| SLS2 (5^th^) | ATCGTCGTGACTGTTTGTAATAGGACAGAGCCCCGCACCAGTCACGACGATGCACTCTTGCCTACCCCAT |
| SLS2 (6^th^) | ATCGTCGTGACTGTTTGTAATAGGACAGAGCCCCGCACCAGTCACGACGATGCACTCTTGCCTAAGCCAT |

Notes: MutDNA is oncogenic single-based mutant of KRAS (G12D), and wtDNA is the wide-type DNA of KRAS. Point mutation in mutDNA and M1 is shown in bold. The additional mismatch site within SLS2 (2^nd^ to 6^th^) was indicated by underline.

**Table S2.** Comparison of the sensing performance of several recently reported ligation-initiated methods for SNP detection.

| **Amplification strategy** | **Detection technique** | **LOD for alleles** | **LOD for target DNA** | **Ref.** |
| --- | --- | --- | --- | --- |
| Ligation-based RNase digestion | Fluorescence | 5% | 10 aM | ^1^ |
| Ligation-rolling circle amplification (RCA) | Microfluidic device | / | 1 fM | ^2^ |
| Ligation detection with double-enhanced electrochemiluminescence | Electrochemiluminescence | 5.9% | 0.8 fM | ^3^ |
| Ligation coupled with strand displacement amplification | Real-time fluorescence | 0.75% | 30 aM | ^4^ |
| Ligation based RCA | Fluorescence | / | 7.79 aM | ^5^ |
| Ligation-initiated LAMP | Real-time fluorescence | 0.1% | 10 aM | This work |

**References**

1 Kim, J. H. PCR free multiple ligase reactions and probe cleavages for the SNP detection of KRAS mutation with attomole sensitivity. *Analyst* **141**, 6381-6386, doi:10.1039/c6an00909c (2016).

2 Heo, H. Y., Chung, S., Kim, Y. T., Kim, D. H. & Seo, T. S. A valveless rotary microfluidic device for multiplex point mutation identification based on ligation-rolling circle amplification. *Biosens Bioelectron* **78**, 140-146, doi:10.1016/j.bios.2015.11.039 (2016).

3 Feng, Y., Sun, F., Wang, N., Lei, J. & Ju, H. Ru(bpy)3(2+) Incorporated Luminescent Polymer Dots: Double-Enhanced Electrochemiluminescence for Detection of Single-Nucleotide Polymorphism. *Anal Chem* **89**, 7659-7666, doi:10.1021/acs.analchem.7b01603 (2017).

4 Zhang, Y., Guo, Y., Quirke, P. & Zhou, D. Ultrasensitive single-nucleotide polymorphism detection using target-recycled ligation, strand displacement and enzymatic amplification. *Nanoscale* **5**, 5027-5035, doi:10.1039/c3nr01010d (2013).

5 Park, K. W., Lee, C. Y., Batule, B. S., Park, K. S. & Park, H. G. Ultrasensitive DNA detection based on target-triggered rolling circle amplification and fluorescent poly(thymine)-templated copper nanoparticles. *RSC Advances* **8**, 1958-1962, doi:10.1039/c7ra11071e (2018).
